# The National Medical Union (Official)

**Published:** 1914-04-18

**Authors:** 


### THE NATIONAL MEDICAL UNION (Official).

Offices : 346 Strand. Tel. : City 8444.

### REPORT OF COUNCIL MEETING.

(*Continued from* THE HOSPITAL, April 4, page 28.)

IN speaking to the resolution that a national medical service is necessary for the genuinely poor classes of the community, Professor Russell said that from the outset he held that the great fault in the National Insurance Act was the wage limit. If the wage limit had been made, say, 20s., the whole profession would have aided the Government in working the Act. A man with a family who had only 20s. a week could not pay for medical attendance to any great extent, and it was the duty of the State to supply medical attendance for such people. In Edinburgh the conception of a national medical service was based upon the fact that there was this large body of poor people unable to pay for medical attendance.

The poor, who up to the time of the passing of this Act had been attended largely by charity, constituted only one-fourth of the persons who were swept into the Insurance Act, according to Mr. Lloyd George's own figures. Those were the people for whom the Government ought to have provided medical attendance, and not only for them, but for their wives and families. The twopence paid by the Government for each person at present insured would have provided a sum of eightpence a week for the genuinely poor persons, calculating them at one-fourth of the present insured persons, and this would have provided for them without taking anything from them or their employers. If the present Government had been wise enough to have done this they would have conferred a great benefit on the community. It was upon these lines that a national medical service was required, and on that basis it would have been supported by the whole profession in Edinburgh. They did not go beyond that—simply to supply the poor in this way, and to take them out of the old region of pauperism. If the National Medical Union adopted a definite policy on this point it would carry with it a great amount of moral support from the public. The public would realise that the Union was not out for spoil. He could imagine medical men devoting their lives to such a service as whole-time men, and that they would obtain as much respect as men at the very top of the profession now enjoy. Make the wage limit 20s. a week or 25s., but let us begin at a point where we all agree.

Mr. Wright thought that medical attendance on all earners of wages above the fixed limit should be provided on the lines of private practice, but that there must always be a class who are unable to make private arrangements with their doctors, and for them a system of national medical service in some form will be required. He therefore moved as an amendment that the resolution should read: "That a national medical service is necessary for the genuinely poor classes of the community, and for them only." This was seconded by Dr. Burgess and carried.

[*Note.*—The report of the meeting published in last week's issue and containing remarks by Professor Russell and Mr. Wright was unfortunately taken from an uncorrected copy. It is, therefore, repeated above in the correct form.]

[A letter on "Non-Panel Men and the Medical Directory" appears on page 77.]

---

AT the meeting of Council on March 21 it was resolved to publish the following correspondence between the Hampstead Non-Panel Association and the General Medical Council, in order that the attitude of the General Medical Council towards advertisement of names, addresses, and times of attendance of doctors practising on the panel might be made clear:—

### HAMPSTEAD NON-PANEL ASSOCIATION.

January 30th, 1914.

The Registrar, General Medical Council.

DEAR SIR,—I am instructed to forward to you a copy of the *Hampstead Advertiser* for January 8th, 1914, in which on page 11 appears an advertisement of the names and addresses and times of attendance of doctors practising on the panel in Hampstead.

This has been complained of and brought to the notice of this Association, and as it is a breach of the rule against advertising, I am requested to ask you to be good enough to submit the matter to the Disciplinary Committee of the Council at the earliest possible date.—I am, yours faithfully, E. ARTHUR DORRELL.

### GENERAL MEDICAL COUNCIL.

January 31st, 1914.

E. Arthur Dorrell, Esq., F.R.C.S.,
7 Cannon Hill, West Hampstead, N.W.

DEAR SIR,—I have received your letter of yesterday, but if you wish to make a complaint it will be necessary, in accordance with the Standing Orders, for the facts to be set forth in the form of a statutory declaration.

It seems to me that you would have to prove that the gentlemen mentioned in the newspaper notice were in some way responsible for its insertion. In view of the fact that the names of those on the panels are put up in the Post Offices, and that, clearly, the insured persons must be furnished with information as to what doctors they may consult, I venture to suggest that it is hardly likely that a complaint of this kind could be sustained. However, if you send one in as prescribed it will be my duty to lay it before the authorities of the Council.—Yours faithfully, NORMAN C. KING, Registrar.

### COPY OF STATUTORY DECLARATION.

February 19th, 1914.

1. That I am Secretary to the Hampstead Non-Panel Association, and that I make this statutory declaration on behalf of the said Association.
2. That the names of certain medical practitioners, together with their professional addresses and the hours at which they see patients at these addresses, appeared in a weekly journal, called the *Hampstead Advertiser*, to wit, in the issue of this paper dated January 8th, 1914.
3. That these practitioners are on the panel for the Insurance District of Hampstead.

4. That the said weekly journal is gratuitously distributed to large numbers of householders in Hampstead.

5. That a copy of the said advertisement referred to in paragraph 2 appears in a publication called the *Hampstead Guide and Almanack*, published and printed by the printers of the aforementioned journal.

6. That, to the best of my knowledge and belief, none of the medical practitioners whose names appear in the said advertisement has taken any steps to disclaim responsibility for the publication of this advertisement or to prevent a repetition of such publication.

GENERAL MEDICAL COUNCIL.

February 23rd, 1914.

E. A. Dorrell, Esq., F.R.C.S.,
7 Cannon Hill, West Hampstead.

Dear Sir,—With reference to the declaration sent in by you on the 20th instant, I am directed to say that, before the complaint can be brought before the Penal Cases Committee, it would be necessary for declarations to be sent in setting forth specifically in the case of each individual person that the advertisement was of an objectionable character, and that it was inserted for the purpose of procuring persons to become their patients. It would be useful also to show that the advertisement was inserted with the knowledge and consent of those concerned. I return your declaration herewith.—Yours faithfully, Norman C. King, Registrar.

HAMPSTEAD NON-PANEL ASSOCIATION.

March 5th, 1914.

The Registrar, General Medical Council.

Dear Sir,—As regards the objections raised by you in your letter of February 23rd, I wish to point out that it is emphatically one of those cases to which what is called in legal parlance the principle *Res ipsa loquitur* applies. Surely an advertisement containing the names of medical men, together with their professional addresses and their hours of attendance, is an invitation to the public to consult these doctors at such times and places. The inference, I claim, is so obvious that it does not require any evidence on my part to support it, but raises a strong presumption, which the parties complained about ought to be called upon to rebut. And this presumption is further materially strengthened by the fact that the gentlemen in question took no steps to disavow responsibility for the advertisement, in circumstances in which the ethics of the profession made it imperative for them to dissociate themselves from such public advertisement.

In conclusion, I wish, however, to add that the object with which the Hampstead Non-Panel Association instructed me to lodge this complaint, was not so much the desire to see disciplinary sanctions enforced on the offenders as their wish to elicit from the General Medical Council a pronouncement which would effectively stop for the future a repetition, here and elsewhere, of what they consider a flagrant breach of a rule hitherto enforced, not only by the General Medical Council, but also by the best traditions of the profession.—I am, yours faithfully,

E. Arthur Dorrell, Hon. Sec.,
Hampstead Non-Panel Association.

GENERAL MEDICAL COUNCIL.

March 10th, 1914.

E. A. Dorrell, Esq., F.R.C.S.,
7 Cannon Hill, West Hampstead.

Dear Sir,—With reference to your letters of the 30th January and the 19th of February I am directed to say that your statutory declaration contains nothing on which the Council can proceed against any of the practitioners. It is not alleged that any of them are aware of or has paid for the advertisement; or that the advertisement is of an illegitimate character, inasmuch as it may have been simply copied by the newspaper (which is supplied gratuitously) from the legally published list which appears in Post Offices, etc.

If you can furnish evidence in supplement to supply these deficiencies I will again lay the matter before the authorities of the Council.—Yours faithfully,

Norman C. King, Registrar.

### The Medical Benefit "Surplus Funds."

The question of the disposal of the funds accumulated in respect of insured persons in London who have declined to choose a panel doctor was once more under consideration at a special meeting of the London Insurance Committee on April 2.

One member of the Committee had attended prepared to move for the distribution of the medical benefit funds forthwith among the panel doctors, a proposal which, no doubt, would have received a measure of support from those present. The transaction of the business was, however, prevented by the receipt at the last moment of counsel's opinion. This, on being read out, once more makes it clear, and in no uncertain terms, that "nothing can be distributed to the practitioners unless the practitioner is by his contract entitled to something, and then he can get only what he is entitled to by his contract." Counsel further stated that, in his opinion, "any member of the Committee who sanctioned the unauthorised expenditure of the money would be personally liable to make it good."

In the face of such legal advice, scarcely less emphatic than that given more than six months ago to the same Committee, it is astonishing that a body which must contain at least some members with business capacity, and which has voted itself payment from public funds for time spent in committee, should waste that time and draw that public money in prolonged efforts to argue that the panel doctor is entitled to payment for insured persons who are neither on his list nor desire to be forced upon it.

Doubtless the utilisation of the surplus medical benefit funds as "largesse" to attract and retain doctors on the panel would have helped to bolster up the tottering panel system; for it will be remembered that Mr. Masterman, in the recent electioneering campaign before his rejection by Bethnal Green, took upon himself to promise a deputation of the panel doctors that the funds would be so disposed of. But now that common sense has been again supported by legal opinion, it is to be hoped that the last has been heard of this proposal. For some eight or nine months the London Insurance Committee has shown an inclination to deal with the problem in a way which it now seems would be illegal. The interesting question now is—What will happen in other county committees where the funds have already been illegally handed over to the panel doctors?

**(Continued on p. 3 of the "Institutional Worker.")**

